# Tri-State Medical Society of Alabama, Georgia and Tennessee

**Published:** 1891-11

**Authors:** 


### TRI-STATE MEDICAL SOCIETY OF ALABAMA, GEORGIA AND TENNESSEE. THIRD ANNUAL MEETING HELD IN CHATTANOOGA OCTOBER 27TH, 28TH AND 29TH.

FIRST DAY.

Society called to order by vice president E. T. Camp.

Opened with prayer by Rev. D. Van Price.

The committee on Necrology offered a set of resolutions on the deaths of Drs. T. P. Garry of Florida, W. B. Wells and W. P. Craig, both of Chattanooga, which were adopted.

After reports of committees and the transaction of some miscellaneous business, Dr. E. E. Kerr reported a case of Neuro-Mimetic hip trouble and presented the patient.

This was a case in which the diagnosis of gonorrhoeal rheumatism had been made but he was unable to see the case in that light. The nervous symptoms and the family history indicated a nervous element and there was an hysterical element in the case. A partial cure was effected by suggestion, but the patient still walked on his toe, for which he could see no reason as there was no shortening nor tenderness about the hip, or other signs indicating organic disease.

Dr. Trippe said that he had treated the case before Dr. Kerr. The patient had had gonorrhœa four weeks before he saw the case. There was increased temperature (102 degrees to 104 degrees) preceded by two very severe chills, cystitis and the typical picture of gonorrhoeal rheumatism, although an hysterical element was recognized in the case.

Dr. Reeves thought the case one of involvment of the cord in which there was an attack of gonorrhoea, and that this set up a new train of reflexes. He called attention to the fact that every discharge from the meatus was not a gonorrhoea, and as a test he stated that the discharge from a specific case was acid from a non-specific alkaline.

Dr. J. B. Cowan thought that the history as given by Dr. Trippe indicates some specific trouble,and if most of the members had seen the case it would have been diagnosed as gonorrhoeal rheumatism. Where we have such a specific trouble and a history of masturbation as here we would expect some hysterical symptoms. Dr. H. Berlin said a man may have an hysterical joint just as a woman may have the globus hystericus.

Dr. Drake from the history and an examination and from the history thought the case one of gonorrhoeal rheumatism.

Whenever there is a pain there must have been a cause past or present.

Dr. Kerr had nothing to say as to the condition before he saw the case.

He agreed that where there were neurotic symptoms it was a difficult task to make a diagnosis. He had brought the patient so as to find out how to make him stop walking on his toe, for which he could see no reason.

AFTERNOON SESSION.

President Robert Battey presided.

Dr. E. T. Camp of Gadsden, Ala., read a pager on

THE SUMMER DIARRHOEA OF CHILDREN, in which he gave as the causes 1st, improper food, 2nd, high temperature, 3d, micro-organisms. In some cases there was a neurotic element. He reported one case where the diarrhoea was cured by circumcision, there being no change in the other treatment.

Dr. Battey asked if any of the members had any experience that would confirm the views of the writer that the prepuce might keep up the diarrhoea.

Dr. Gahagan had a case of persistent diarrhoea in which there was an elongated prepuce. He would circumcise the case and report next year.

Dr. Cowan had not noticed that male children were more subject to diarrhoea than females. There was often fault in the diet both as to quality and especially quantity.

Dr. Berlin called attention to the fact that the Jewish children have diarrhoea as frequently as the Gentiles, and could see no connection between a stomach loaded with bacteria and an adherent prepuce.

Dr. Reeves said that there was no specific cause or specific origin.

Dr. J. L. Atlee confirmed the experience of Dr. Camp. He had seen cases in which after circumcision the diarrhoea began to improve with no change in the other treatment.

In closing Dr. Camp said that he had reported but one case in his paper, but that he had seen a number of others in which there was a like result.

Dr. George Wiley Broome of St. Louis, read a paper on

REPORT OF A SUCCESSFUL CASE KALPOO-HYSTERECTOMY, INCLUDING A BRIEF REVIEW OF THE PRESENT STATUS OF THE OPERATION, in which he advocated the operation in all cases of epithelioma or carcinoma of the cervix or of the body of the uterus regardless of the extent of the disease.

Amputation of the uterus should never be performed.

Dr. Davis' experience had been that these cases when sent to him were too far advanced to justify an operation. He had not been convinced where but a limited part of the cervix was involved that an amputation was not as good as the radical operation. Many cases were morphine eaters and the condition of the intestinal tract was one of importance.

Dr. Berlin thought the total extirpation was better than high amputation. When the disease had passed beyond the uterus it was too late to amputate in any way.

Dr. Battey had grave doubts as to the advisability of the operation in the early stage, the diagnosis was difficult. In some of the cases sent him as cancerous cures were effected by the application of iodine, etc.

Of the cases reported cured he had grave doubts as to the diagnosis.

On the other hand there were many deaths after the operation, if not immediately, within a short time. As in the case of Gov. Hill, many will not consent to an operation until a malignant growth has advanced beyond the stage when it can be removed.

Dr. Key preferred the clamp to the ligature. Early diagnosis is of importance,and this can only be made by an expert pathologist, and as soon as made the uterus should be removed.

NIGHT SESSION.

Addresses of welcome were made by Dr. J. R. Rathmell, President of the Chattanooga Medical Society, and Col. Garnett Andrews, Mayor of the city.

Dr. Robert Battey responded on behalf of the society.

Dr. Geo. R. West reported

TEN CASES OF LAPAROTOMY, with one death. Three were for the removal of diseased ovaries and tubes, one for the cure of oophoro-epilepsy, one for the removal of ovarian cyst, three for the relief of symptoms caused by uterine fibromata, two exploratory incisions. Of the nine recoveries, six were perfect cures, three partial cures from incomplete operations.

Dr. Davis said that it was the improved technique that gave success in these operations, which required not only book knowledge but special training.

Dr. Broome advocated early operation. He insisted on sterilizing the instruments and endorsed Arnold's sterilizer. Morphine should never be given after a laparotomy.

Dr. Reeves felt grateful to the author for his remarks on conservatism. He quoted Wier Mitchell, who said that in his experience he had sent thirteen cases to the surgeon. Five of these were not improved. Dr. Gardner had said that the majority of cases operated on were not any better five years after the operation.

SECOND DAY.

Opened with prayer by the Rev. J. W. Bachman.

After some miscellaneous business Dr. Robert Battey addressed the association on

OVARIOTOMY; ITS USE AND ABUSE.

He said that the fundamental idea in the operation he had devised was to produce rest. The difficulty of curing many chronic diseases lies in the fact that rest is an impossibility, as with the heart, rest means death. Rest is an impossibility with an ovary.

The objects of the operation are,

1st. The prolongation of life. Years ago Sir Spencer Wells said that he had added 3,000 years to the sum of human life. Now it is probably double that.

2nd. The restoration of a disordered mind. There is a prejudice against the operation, owing to the fact that cases have not been properly selected and alienists want the ovariotamist to cure their cases after they have exhausted every other means of cure, when it is often too late.

Dr. Goodell asserted that an insane woman had no business with children.

Dr. Battey would hardly go so far.

3rd. The cure of epilepsy. As in the case of insanity there should be some connection between the epilepsy and the ovaries. It does not follow that because a woman has epilepsy that her ovaries should be removed. Here Dr. Goodell had good results.

4th. The relief of intolerable pain. Especially when the pain has a tendency to produce that detestable habit, opium eating, a habit little short of insanity. Where the habit has been formed the operation will cure the case if the woman can break the habit.

One of the abuses of the operation is to perform a single operation for the sake of the notoriety it would bring. This ought to be a specialty as much as the eye. Success depends on the skill of the operator, which can come only from experience. It depends also on native ability, and every man should study his natural talents in the light of statistics and choose the field where he is most successful.

The operation of ovariotomy to stop child-bearing is a detestable practice. The operation should never be done without ample consultation, first, to protect the physician, second, in the interest of the profession at large, third, in the interest of the patient.

Dr. Davis thought that as much could be done by simply incising the muscle as by a normal ovariotomy. The operation has no place in the treatment of nervous diseases.

Dr. Broome suggested that as it was well known that ovariotomy produced atrophy of the fibroid tumors by cutting off the blood supply, therefore ligation of the uterine artery might produce as good results.

Dr. Wilson advocated the operation in cases of mania, did not believe that insane women should have children.

To confirm Dr. Battey's views, Dr. Cowan reported a case of epilepsy cured by the operation.

Dr. Battey in closing, gave the indications for the operation, viz:

1st. The case must be desperate. 2nd. It must be incurable by ordinary means. 3rd. There must be a reasonable hope of cure.

In the last two years he had advocated the removal of senile, diseased ovaries for the cure of insanity, citing cases.

Dr. W. E. B. Davis, of Birmingham, Ala., read a paper entitled

TREATMENT OF INFLAMMATIONS ABOUT THE HEAD OF THE COLON, in which he said that cases must be selected for the operation, important symptoms must not be masked by the administration of opium.

More reliance should be placed on regional tenderness than on the temperature. An inflammation about the head of the colon is always an appendicitis, the involvment of surrounding tissues being secondary. Early operation is necessary.

Dr. Cunningham was of the opinion that the whole be rewritten. The peritoneum is always involved to a limited extent.

Dr. Shimwell said that the temperature may not be increased, and related a case confirming the statement. There is no rule when to operate, each case must be judged on its own merits.

AFTERNOON SESSION.

Dr. Karl von Ruck of Asheville, N. C. read a paper on

THE CURE OF TUBERCULOSIS ON THE PRINCIPLE OF NUTRITION, in which he said that the diagnosis with the microscope could not be made in the early stage. No one measure should be relied upon in the treatment. He was surprised that greater harm had not been done by the large doses of tuberculin that had been used. In the early stage the treatment was often inefficient when the cases could be cured. Climate was of importance, and all measures that could benefit the patient should be employed.

Dr. Reeves advised the use of the microscope in all cases to confirm the diagnosis; if it be not tuberculosis it is syphilis. Primarily, the disease is due to lymph stasis.

Dr. von Ruck said that in the early stage there was no sputum.

Dr. J. C. Shepard, of Winchester, Tenn., read a paper on

MILK SICKNESS, stating that the disease existed only in a limited area; that it was contracted from the cow. The poison seemed to be neither animal nor vegetable, but mineral. The disease called trembles in the cow resembled lead poisoning in man.

Dr. Cowan said that the subject was of so much importance that the government had offered a reward for the discovery of the cause. He had seen one case, and thought at first it was lead or cobalt poisoning.

Dr. Reeves said that the bacteria had been found; that they were spirilli, for which quinine was the best remedy.

Dr. J. B. Murfree, of Murfreesboro, Tenn., read a paper on

THE NECESSITY OF ASEPSIS IN PRIVATE OBSTETRICAL PRACTICE.

He advanced the idea that it was more necessary to protect the wounded surface here than in an open wound. The decreased mortality in hospital practice, he thought due to the use of antiseptics. In private practice cleanliness was necessary, and sometimes antiseptics, especially should the hands be clean, and the examinations be as few as possible.

Dr. Baxter endorsed the paper in the main, but thought that in private practice the danger of infection ten times as great as in hospital practice. The nurses should be watched, as they know nothing of surgical cleanliness.

Dr. Shimwell thought the injury to the mother was a factor in these cases that was overlooked. Wherever there has been a post mortem, great injury to the tissues had been found.

Dr. Cowan thought the great secret was cleanliness, but that antiseptics have their place.

In a large number of cases observed, Dr. Wilson had not found the result any better with antiseptics than with simple cleanliness with sterilized water. The results were as good where the patients were aggregated as where they were segregated. Vaginal irritation was not necessary, for the cases did as well by simply washing the vulva.

Dr. Cunningham thought with Dr. Shimwell, that the result was often due to traumatism. He always uses the Crede method of expelling the placenta.

NIGHT SESSION.

At 8 o'clock p. m. an elegant reception was tendered the members at the residence of Mr. and Mrs. W. R. Wilson, 229 East Terrace.

THIRD DAY—MORNING SESSION.

Opened with prayer by Rev. Robert J. Willingham.

Dr. W. G. Bogart, of Chattanooga, read a paper on

LACERATED CERVIX.

He advocated the operation only when there were troublesome symptoms produced by the laceration, and other measures fail. He described the operation mainly as laid down by Skeene. The causes of failure were, imperfect preparation of patient, imperfect operation, or imperfect after treatment.

Dr. Camp said that it was necessary to remove all the cicatrical tissue. Silver sutures the best. Douches not necessary. He does not endorse the use of ergot after delivery.

Dr. Davis said the paper presented the present status of the operation. The condition requiring it could be prevented by proper attention after confinement. Ergot is of use after confinement, not only to cause contraction of the uterus, but it also closes the mouths of the small vessels and lessens the danger of septic poisoning. He examines all of his patients six weeks after confinement, if possible. In subinvolution, the faradic current is of value, despite the assertions of many that electricity was of no use in gynæcology.

Dr. Reeves had gotten good results in these cases by supporting the womb with a Fowler pessary, and had cured some by this means. He gave minute doses of ergot after confinement.

Dr. Bogart, in closing the discussion, said that he gave support to the uterus in these cases, but that he preferred to do this with medicated lamb's wool tampons instead of using a hard rubber pessary.

Dr. W. G. Drake, of Chattanooga, presented a paper on

THE PHYSIOLOGY AND CHEMISTRY OF THERAPEUTICS.

In this he maintained that the infectious diseases are caused by ptomaines or toxines evolved by bacteria in the body. He proclaimed that "chemical antagonism" was "the safest, the most scientific and most rational means of cure," rather than that of "physiological antagonism." He argued that all bacterial toxines had an antidote for which he should look. The tendency was to return to specific medication along more scientific lines. The age demands rational medicine.

Dr. Purdon called attention to the fact that the antiseptics were used thirty years ago empirically, for he had used the permanganate of potash in cholera; he had also used the peroxide of hydrogen.

Dr. B. T. Shimwell, of Philadelphia, read a paper on

ARTIFICIAL ANUS VS. ANASTOMOSIS,

Dr. John E. Purdon of Cullman, Ala., read a paper on

THE CONSERVATION OF ENERGY IN MODERN PHYSICS, in which he claimed that in the face of established facts of mental and physical action at a distance, nothing was left to the physiologist but to acknowledge the existence of an extra-muscular mode of the externalization of energy in relation with conscious or sub-conscious will and design. He held the opinion that the ether of space had its physiological as well as its physical side and that as the reservoir of the work doing power of the universe it bore a relation to the Universal Life analogous to that which the blood and the nervous system held to the individualized spirit. He based his theory of an ethereal nervous medium upon the results of his own sphygmographic researches which showed the similarity of the pulse traces of individuals *en rapport*, during extraordinary manifestations of energy, such as "knockings" and telepathic influence.

Dr. Purdon deposited publicly with the secretary the photographs of a selected set of pulse tracings, taken by himself, in illustration of the above view, and claiming the absolute originality of the method for himself.

Dr. Cowan said that the grandest result of energy was thought; by the arrangement of matter by the correlation of force we have this power.

This we derive from solar force.

Dr. Cunningham thought that we knew nothing about the matter.

Dr. Drake took issue with Dr. Cowan that the original force was solar, for energy existed before the sun was made and came from the Deity. To this Dr. Cowan assented.

Dr. J. P. Stewart of Attalla, Ala., read a paper on

EVOLUTION FROM A SCIENTIFIC STANDPOINT, in which he advocated the doctrine from scientific considerations.

Dr. Drake said that the re-productive energy in the human ovum was the unseen hand of God moulding its protoplasm into a perfect form.

Dr. Purdon said that man belongs to a different class from the lower animals. Evolution is true as a formula, as a partial formula.

AFTERNOON SESSION.

Dr. Henry Wm. Blanc of Sewanee, Tenn., gave his experience in

A REVIEW OF FIVE YEARS DERMATOLOGICAL PRACTICE IN NEW ORLEANS.

He reported 2013 cases seen in public and private practice. Twenty-five per cent. were eczema, elsewhere the per cent. is 30 or 35 Epithelioma in the form of rodent ulcer, figured conspicuously in the report. A large number of leprosy cases were reported, many of these cases were of foreign birth or children of foreigners. The author believes in the contagiousness of leprosy, but thinks that in many of his cases the disease was contracted from some animal source, as in eating raw meat or in preparing meat for the table.

Dr. R. M. Cunningham read a paper on

CROUPOUS PNEUMONIA.

A paper was read by Dr. Y. L. Abernathy of Hill City, Tennesse, on

DOCTORS.

Dr. W. P. McDonald of Hill City, read a paper entitled

LEGISLATION, which was not discussed, as it dealt with matters of a political nature.

Dr. W. C. Townes read a paper on

ANGINA PECTORIS, in which he gave as the conditions in the disease, first, pseudoangina pectoris; second, that form in which there is sclerosis of the coronary arteries; third, where there is valvular disease. The treatment depends on the cause. In the first form we have a neurosis and we correct anything we find at fault with any of the organs; secondly, we give tonics, potass. iodide, arsenic nitrites; thirdly, we prescribe during the attacks such drugs as amyl nitrite, chloroform and opium.

Dr. Drake thought angina pectoris a symptom rather than a disease, sometimes the result of organic lesions, but often merely a cardiac neuralgia. He uses nitro-glycerine with atropia for the pain.

Dr. Purdon gives as routine treatment the salicylate of soda where it is caused by cold (lowering of temperature.) This is combined with stropanthus to prevent relapses.

Dr. Camp believes it to be due to a rheumatic diathesis and uses chloroform by inhalation.

Dr. Wert would be afraid to give chloroform owing to the pathology.

Dr. Purdon said that by no means must electricity be used.

Dr. Baxter did not think chloroform specially dangerous, and cited cases.

Dr. Cunningham believes in giving atropia and nitrite of amyl. He did not consider an intermittent pulse to contra-indicate chloroform.

Dr. Townes closed by saying that he did lay much stress on the above treatment.

NIGHT SESSION.

Dr. E. H. Kuykendall of Chattanooga, read a paper on

BROMIDE OF ETHYL AS AN ANAESTHETIC, advocating its value and safety when given for short operations (one minute) and in dose of not over a drachm. It is given free from air, anæsthesis is complete from one-half to one minute. The effects last about two minutes, when the patient wakes as from a natural sleep. Nausea is seldom produced.

Dr. Davis said that one accustomed to give ether was not safe to give chloroform, and it might be so with this, the deaths may have been due to faulty administration. Nitrous oxide was a rapid anaesthetic and was considered the safest

Dr. Smith suggested that if the doctor would give the number of cases observed by him that it would be of interest.

Dr. Berlin said that an objection was the odor. He related two cases of death from the drug.

Dr. Gahagan asked Dr. Kuykendall for the mortality, how anæsthesia was produced, and the antidote.

Dr. Kuykendall replied that there had never been a fatal case unless the administration was prolonged. Dr. Chisholm had used it in 300 cases without a bad result. So far as he knew there had been but two deaths.

He did not know how it kills or how it produces anaesthesia. The antidote is the same as in threatened death from chloroform.

Dr. Willis F. Westmoreland, of Atlanta, discussed

BRAIN SURGERY, saying that the surgeon had gone into the brain, where physiologists had said they could not go. An exploratory incision into the brain substance was just as justifiable as in laparottomy. In abscess and tumors there has never been a cure without operation. Where the incision has been thorough the results have been good. The safeguard is antisepsis, without which there is uncertainty. In operating, the ventricles must be avoided.

Dr. Drake argued that the surgeon had never gone farther than the physiologists had mapped out for them. They dare not invade the 4th ventricle in the vicinity of the respiratory center.

Dr. Westmoreland reminded Dr. Drake that it was not due to the physiologists, but to the fact some years ago a man had recovered after a crowbar had gone through his brain.

Dr. Berlin related a case of insanity coming on after an injury to the skull, cured by an operation, with a relapse and a second cure by the same means.

Dr. Crumley believed that all functions were localized, some areas can be invaded, others cannot.

Dr. Cunningham said that most of these cases would die without operation and that the surgeon was justified in doing anything that offered the least hope.

Dr. Stewart reported a case of brain surgery where the whole frontal bone was taken away.

Dr. Westmoreland said that to Dr. Briggs was due the credit of being one of the first to do this work. The success depends largely on drainage, and it may be necessary to make a counter opening.

The following are the officers for the ensuing year:

President, W. E. B. Davis, Birmingham, Ala.

Vice-Presidents :

D. H. Howell, Atlanta, Ga.

J. C. Shapard, Winchester, Tenn.

J. P. Stewart, Attala, Ala.

Secretary, Frank Trester Smith, Chattanooga.

Treasurer, B. S. Wert, Chattanooga.

Recorder, W. L. Gahagan, Chattanooga.

Councillors, J. B. Murfree, A. B. Frix, John E. Purdon, G. W. Drake, J. W. Clements, E. T. Camp.

---

The Mississippi Valley Medical Association held its 17th annual session at St. Louis, October 14th, 15th and 16th, 1891, President Dr. C. H. Hughes, of St. Louis, in the chair. The attendance was large, the papers numerous and valuable. Dr. I. N. Love, the incomparable Chairman of the Committee of Arrangements, and his able assistants, deserve unstinted praise for their provision of receptions, rides, dinners, suppers, banquets, fine weather and full moon. Dr. C. A. L. Reed, of Cincinnati, was elected President, Dr. E. S. McKee, Cincinnati, re-elected Secretary, Dr. C. S. Bond, Richmond, Ind., 1st Vice-President, Dr. J. H. Stucky, Louisville, 2nd Vice-President, Dr. Joseph Ransohoff, Cin., Chairman Committee Arrangements. Place of meeting, Cincinnati, October, 1892.

